# Supplementary material for: Optimizing the thermoelectric performance of graphene nano-ribbons without degrading the electronic properties
Source: Sci Rep. 2017 May 24;7:2313. doi: 10.1038/s41598-017-02230-0 (PMC5443772; doi:10.1038/s41598-017-02230-0)
Supplement: Supplementary file 1 — Supplementary Material [file 41598_2017_2230_MOESM1_ESM.pdf]

Optimizing the thermoelectric performance of graphene nano-ribbons without degrading the electronic properties

Van-Truong Tran[1], Jérôme Saint-Martin[2], Philippe Dollfus[2] and Sebastian Volz[1]

[1]EM2C, CentraleSupélec, Université Paris Saclay, CNRS, 92295 Châtenay Malabry, France
[2]C2N, Université Paris-sud, Université Paris Saclay, CNRS, 91405 Orsay, France
van-truong.tran@ecp.fr and sebastian.volz@ecp.fr

Supplementary information for methods

Although the first nearest neighbor Tight Binding (TB) calculation has been extensively used in many works to investigate electron properties, it has been shown that TB models involving up to the third nearest neighbor (3NN) interactions fit ab initio calculations more accurately. [1,2] Within this sophisticated calculation, the overlap matrix was also added, providing an optimized description of TB calculations in good agreement with ab initio results and suitable to large devices. [1,2]

In the present work, the 3NN TB model was employed for electron study using parameters taken from the work of Reich. [1] The Hamiltonian of the whole system can be written generally as

$$H\psi = ES\psi, \qquad \text{(S1)}$$

where H, S, E and ψ are the Hamiltonian, the overlap matrix, the eigen energy and the wave function, respectively. The matrix elements are calculated as follows: $H_{ii} = \varepsilon_i$ is the on-site energy at *i*th site, $S_{ii} = 1$ because the orbital wave function at the *i*th site is orthogonal itself, $H_{ij} = -t_{ij}$ and $S_{ij} = s_{ij}$ with t_{ij} and s_{ij} referring to the hoping and overlap parameters, respectively, between atoms at the *i*th and *j*th sites. Each couple of parameters $\{t_{ij}, s_{ij}\}$ must be selected as $\{t_0, s_0\}$, $\{t_1, s_1\}$ or $\{t_2, s_2\}$, i.e. as the first, second and third nearest-neighbor parameters, respectively, depending on the distance between atoms *i* and *j*.

For phonons, we employed a Force Constant (FC) model involving up to the four nearest neighbor interactions, which has provided a precise reproduction of the phonon dispersion of graphene obtained by ab initio methods and experimental measurements. [3] The force constant parameters were taken from Wirtz's work.[3] The motion equation of Newton's second law,[4] can be rewritten in a matrix form with the Dynamical matrix *D*, which can be seen as the matrix form of the Hamiltonian equation for phonons:[4,5]

$$DU = \omega^2 U, \qquad \text{(S2)}$$

where U is the matrix containing the vibrational amplitudes of all atoms and ω is the angular frequency. Since the coupling between two atoms i and j is described by a 3x3 tensor, the block elements of the dynamical matrix D are finally [5]: $D_{3\times3}^{ii} = \sum_{j\neq i} \frac{K_{ij}}{M_i}$ and $D_{3\times3}^{ij} = -\frac{K_{ij}}{\sqrt{M_i M_j}}$ for $j \neq i$. The tensor coupling between atoms i and j, K_{ij} is defined from the force constant matrix parameters by a unitary in-plane rotation. [4,5]

When introducing substitutional ^{14}C isotopes in the ^{12}C lattice, the electron Hamiltonian was kept unchanged, while the dynamical matrix was adjusted by changing the mass at the positions of the ^{14}C isotopes.

To describe the presence of vacancies, we switched off all hoping or tensor couplings between a vacancy position and its neighbors. The onsite energy and mass at the vacancy positions were also set equal to infinity to ensure that the wave functions or vibrations vanish at the positions of vacancies.

The position of an isotope or a vacancy is defined by 3 indices (n, L, m) where n is the position of the unit cell, with n = 1:N_A, L is either L_1 or L_2, i.e. the line position in the unit cell, and m is the position of the disorder site in the line L_1 (or L_2), i.e. m =1:M.

As equations for electrons and phonons are similar (with $S = \mathbf{1}$ for the case of phonons), we employed in both cases the same method based on the Green's function formalism to compute the transport properties.[6] In practice, the Hamiltonian H and the overlap matrix S were divided into three parts H_L, H_D, H_R and S_L, S_D, S_R (similarly D_L, D_D, D_R for phonons) as the Hamiltonians and overlap matrices of the left contact, device part and right contact, respectively. The coupling terms between the device and the two contacts are denoted H_{DL}, H_{DR}, S_{DL}, and S_{DR} (D_{DL}, D_{DR} for phonons). The Green's functions of the structure for electrons write:

$$G = \left[E^+ . S_D - H_D - \Sigma^s{}_L - \Sigma^s{}_R \right]^{-1}, \qquad \text{(S3)}$$

where $E^+ = E + i.\eta$ and η refers to a positive infinitesimal number added to the energy to avoid the possible divergence of Green's functions and

$$\begin{aligned} \Sigma^s{}_L &= \left(E^+.S_{DL} - H_{DL}\right) G^0{}_L \left(E^+.S_{LD} - H_{LD}\right) \\ \Sigma^s{}_R &= \left(E^+.S_{DR} - H_{DR}\right) G^0{}_R \left(E^+.S_{RD} - H_{RD}\right) \end{aligned} \qquad \text{(S4)}$$

define the surface self-energies contributed from the left and right contacts. $G^0{}_{L(R)}$ represents the surface Green's function of the isolated left (right) contact. Although Sancho's technique is widely used to compute the surface Green's functions,[7–9] the original method does not include the overlap matrices and a correction was thus applied to this method.[10,11]

For phonon calculation, a similar formalism was applied just by replacing energy E by ω^2 , and H_D, H_{DL}, H_{LD}, H_{DR}, H_{RD} by D_D, D_{DL}, D_{LD}, D_{DR}, D_{RD}, respectively. We also considered that $S_D = \mathbf{1}, S_{DL} = S_{LD} = S_{DR} = S_{RD} = \mathbf{0}$ for phonons.

The size of the device Green's function was reduced using the recursive technique. [8,9] Then electrons (phonons) transmission was computed as [5,6]

$$T_{e(p)} = Trace\left\{\Gamma^s{}_L \left[i\left(G_{11} - G_{11}{}^{\dagger}\right) - G_{11}\Gamma^s{}_L G_{11}{}^{\dagger}\right]\right\}, \tag{S5}$$

where $\Gamma^s_{L(R)} = i\left(\Sigma^s_{L(R)} - \Sigma^s_{L(R)}{}^{\dagger}\right)$ denotes the surface injection rate at the left (right) contact. The electrical conductance, the Seebeck coefficient, the electron and phonon thermal conductance were computed using the Landauer-Onsager's approach [12], i.e.

$$\begin{aligned} G_e(\mu,T) &= e^2.L_0(\mu,T) \\ S(\mu,T) &= \frac{1}{e.T}.\frac{L_1(\mu,T)}{L_0(\mu,T)} \\ \kappa_e(\mu,T) &= \frac{1}{T}.\left[L_2(\mu,T) - \frac{L_1(\mu,T)^2}{L_0(\mu,T)}\right] \end{aligned} \tag{S6}$$

Although the intermediate functions L_n are usually formulated by making use of the Fermi function, we derived a more convenient form for practical use [5]

$$L_n(\mu,T) = \frac{1}{h}\int_{-\infty}^{+\infty} dE.T_e(E).(2K_bT)^{n-1}.g^e{}_n(E,\mu,T), \tag{S7}$$

where $g^e{}_n(E,\mu,T) = \left(\frac{E-\mu}{2K_bT}\right)^n / \cosh^2\left(\frac{E-\mu}{2K_bT}\right)$ is a dimensionless function, which decays very quickly with respect to energy and can be used to estimate the boundaries of the integral (7). We also derived a similar form to calculate the phonon conductance

$$K_p = \frac{K_b}{2\pi}\int_0^{\infty} d\omega.\mathrm{T}_p(\omega).g^p(\omega,T), \tag{S8}$$

where $g^p(\omega,T) = \left(\frac{\hbar\omega}{2K_bT}\right)^2 / \sinh^2\left(\frac{\hbar\omega}{2K_bT}\right)$ and K_b refers to the Boltzmann constant.

Once the electrical conductance, the Seebeck coefficient, the electron and phonon thermal conductances are obtained, the figure of merit ZT is readily computed by the following equation [5,13,14]

$$ZT = \frac{G_e.S^2}{K_e + K_p}.T \qquad \text{(S9)}$$

In some cases, thermoelectric ability of a material can be decomposed into fractional contributions of electrons and phonons separately as $ZT = ZT_e / \left(1 + K_p / K_e\right)$ with $ZT_e = \left(G_e.S^2 / K_e\right).T$ is considered as the electron figure of merit.

Supplementary information for density functional theory (DFT) calculations

In the presence of vacancies, the structure maybe deformed and the electronic properties may be changed. Since relaxation is commonly not included in the tight binding description, to show that physics predicted by tight binding calculations is still relevant, we carried out some DFT calculations in which a full structure relaxation was included.

It is worth to note that tight binding treats the truncation at the edges by considering extended lines of carbon atoms at two sides of the edges to compensate the sp2 dangling bonds of carbon atoms at the edges[15], these two extended lines are considered as hard walls and do not influence on the results of tight binding calculations. Therefore, to make a fair comparison of predictions by tight binding and DFT, in DFT calculations we added hydrogen atoms to Carbon atoms at the edges and where vacancies are present to compensate the sp2 dangling bonds.

We have implemented DFT calculations with the SIESTA code[16] which is based on the localized orbital basis set similarly to that in the tight binding description. Since DFT calculations are very expensive and eventually impracticable for large structures/devices, we therefore only considered structures in which the scattering region contains few unit cells with aim at checking physics in the presence of vacancies. We have also used the Perdew-Burke-Ernzerhof (PBE) exchange-correlation functional within the Generalized Gradient Approximation (GGA).[17] The single Zeta orbital basis set was used and a mesh energy cutoff of 300 Ry was chosen. A Monkhorst-Pack[18] 10×1×1 was used for integrals. Both electrodes and the scattering region of all devices were relaxed with conjugate-gradients (CG) method until the total force was less than 0.04 eV/Angstrom.

In Fig. S1, we show the DFT results for a device of length N_A = 5 unit cells in the active region (scattering region). A ribbon of width M = 11 carbon dimer lines with different positions of vacancies was investigated.

Figs. S1(a1), S1(a2) and S1(a3) show the relaxed structures of the scattering region for a vacancy at position $m = 4$ (group $3i + 1$), $m = 5$ (group $3i + 2$) and $m = 6$ (group $3i$), respectively. As it can be observed clearly, the structures are indeed deformed around the position of the vacancy. To examine the electronic redistribution due to the presence of the vacancy, we calculated transmission profiles using the tranSIESTA module (a part of SIESTA code) which is also based on Green's function formalism. The results are shown in Fig. S1(b1) in which the left panel is the band structure of the electrodes. It is obvious that the blue and green lines indicate that vacancies at positions $m = 3i + 1$ and $3i + 2$ reduce strongly the electron transmissions. The vacancy at position $3i$ also leads to a decrease in transmission, but mainly in the high energy regions while the low energy transmission near the bandgap (first transmission steps in the conduction and valence bands) is almost unchanged.

Thus, DFT results are in agreement with what predicted by tight binding calculations presented in Fig. 5(b) in the manuscript. Using the obtained transmission in Fig. S1(b1), we have calculated the power factor which reflects the electronic contribution to thermoelectric effects. The result is depicted in Fig. S1(b2). Again, the results reveal that the electronic properties are almost unchanged, particularly the highest peak, with the presence of a vacancy $3i$, while other positions of vacancy induce strong reductions.

To see how the vacancies $3i$ influence the electronic properties as the scattering region length increases, in Fig. S2 we show relaxed structures and transmissions for two other devices with N_A = 3 and 7. The results still confirm that the electronic properties in the low-energy region are almost unaltered whatever the length of the scattering region.

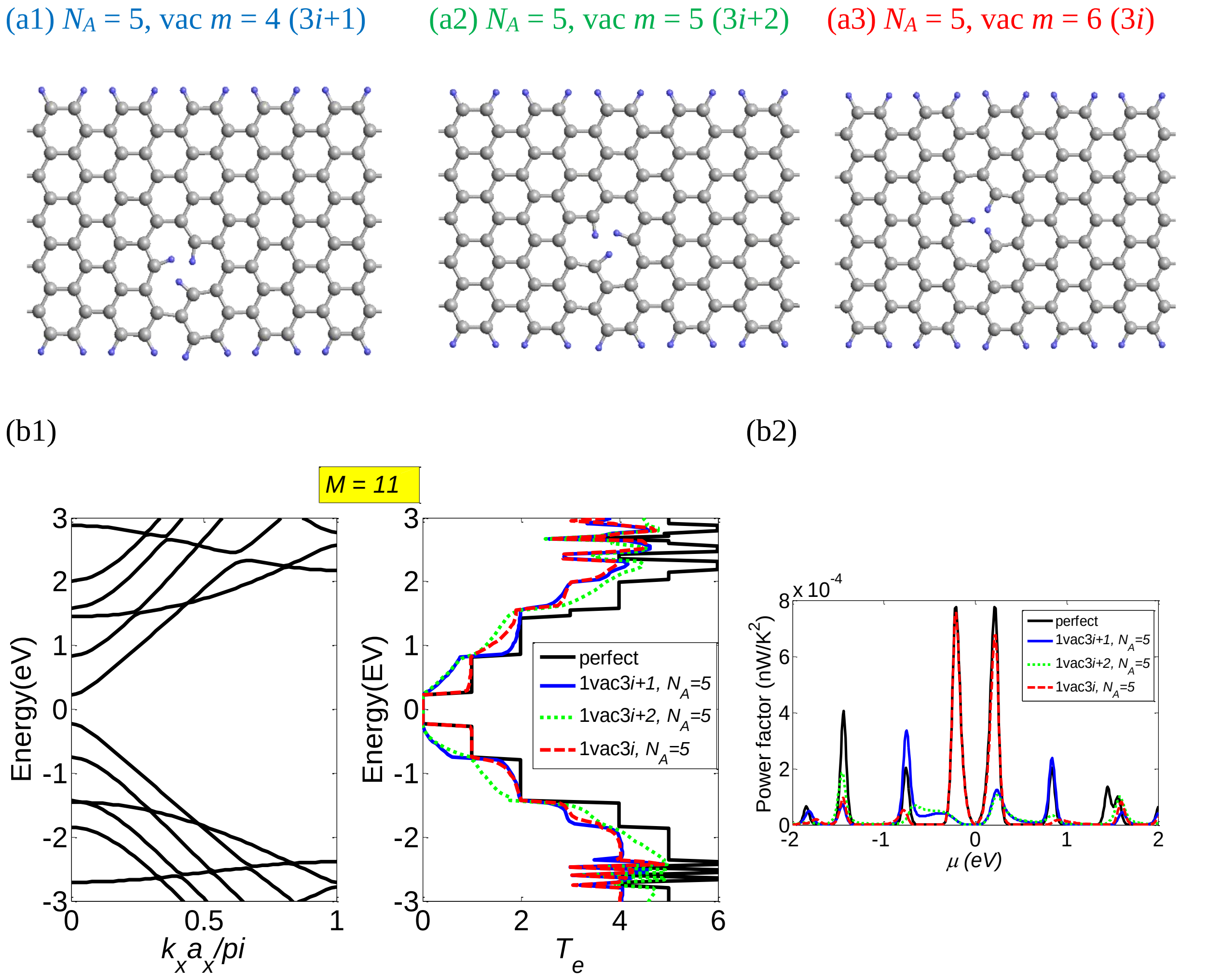

Fig. S1: The relaxed structures of the scattering region of devices of length N_A = 5 unit cells in the presence of one vacancy at position (a1) *m* = 4, (a2) *m* = 5 and (a1) *m* = 6. (b1) The electronic structure of the electrodes and the transmission profiles of the defect structures compared to the perfect one. (b2) The power factor calculated for without and with vacancies.

(a1) $N_A = 3$, vac $m = 6$ (3i)

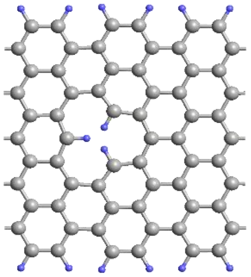

(a2) $N_A = 7$, vac $m = 6$ (3i)

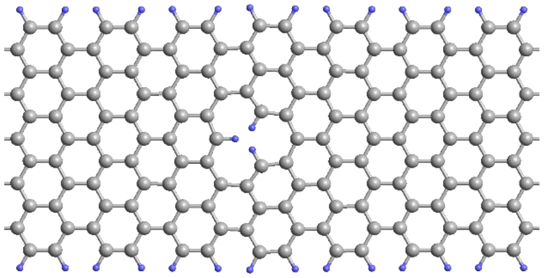

(b)

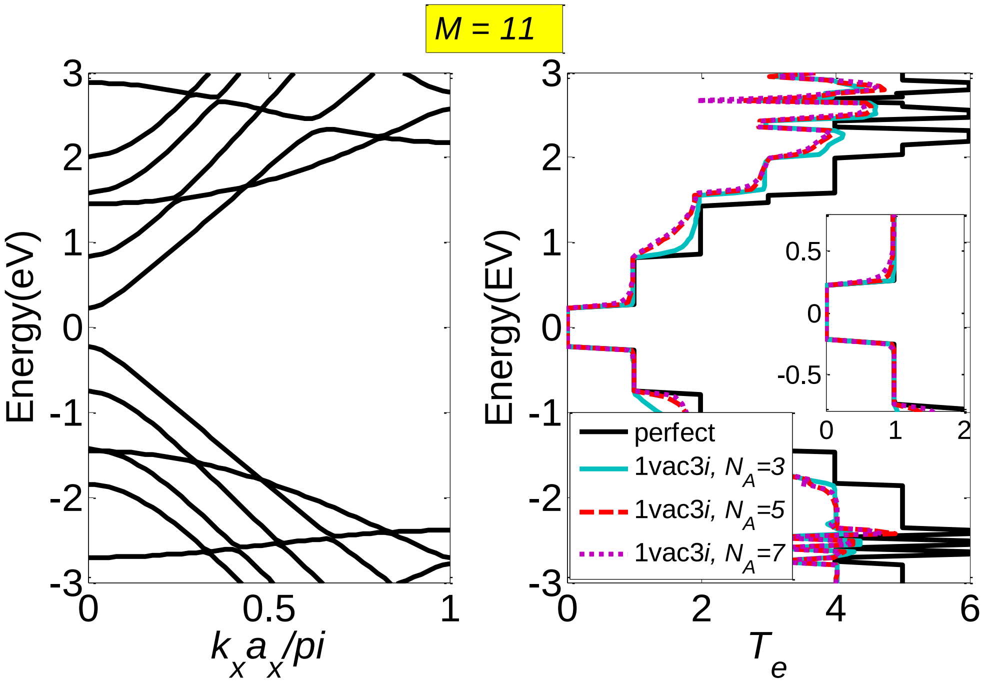

Fig. S2: The relaxed structures of the scattering region of devices of length N_A = 3 (a1) and 7 (a2) in the presence of a vacancy at position m = 6. (b) The electronic structure of the electrodes and the transmission profiles of the structures without and with vacancies.

Supplementary data

Table S1: Fitting parameters for the inverse of the thermal conductivity in the case of isotope doping (Fig. 4b).

	100 K	300 K	400 K	800 K
p_1 (nm.K.nW^{-1})	- 3.0391	- 6.2710	- 6.7673	- 7.3256
p_2 (K.nW^{-1})	3.9162	2.2290	2.0586	1.8518
p_3 (K.nW^{-1}.nm^{-1})	0.0035	0.0047	0.0050	0.0054

Table S2: Fitting parameters for the inverse of the thermal conductivity in the presence of both isotopes and 3*i* vacancies (Fig. 7).

	100 K	300 K	400 K	800 K
p_1 (nm.K.nW^{-1})	-29.6450	-28.3610	-27.9020	-27.2930
p_2 (K.nW^{-1})	6.8577	4.2303	3.9148	3.5301
p_3 (K.nW^{-1}.nm^{-1})	0.0204	0.0237	0.0242	0.0250

References

1. Reich, S., Maultzsch, J., Thomsen, C. & Ordejón, P. Tight-binding description of graphene. *Phys. Rev. B* **66,** 1–5 (2002).

2. Cresti, A. *et al.* Charge transport in disordered graphene-based low dimensional materials. *Nano Res.* **1,** 361–394 (2008).

3. Wirtz, L. & Rubio, A. The phonon dispersion of graphite revisited. *Solid State Commun.* **131,** 141–152 (2004).

4. Saito, R., Dresselhaus, G. & Dresselhaus, M. S. *Physical Properties of Carbon Nanotubes*.

Carbon Nanotubes **3,** (1998).

5. Tran, V.-T., Saint-Martin, J. & Dollfus, P. High thermoelectric performance in graphene nanoribbons by graphene/BN interface engineering. *Nanotechnology* **26,** 495202 (2015).

6. Datta, S. Nanoscale device modeling: the Green's function method. *Superlattices Microstruct.* **28,** 253–278 (2000).

7. Sancho, M. P. L., Sancho, J. M. L. & Rubio, J. Quick iterative scheme for the calculation of transfer matrices: application to Mo (100). *J. Phys. F Met. Phys.* **14,** 1205–1215 (2000).

8. Do, V.-N. Non-equilibrium Green function method: theory and application in simulation of nanometer electronic devices. *Adv. Nat. Sci. Nanosci. Nanotechnol.* **5,** 33001 (2014).

9. Lewenkopf, C. H. & Mucciolo, E. R. The recursive Green's function method for graphene. *J. Comput. Electron.* **12,** 203–231 (2013).

10. Wu, Y. & Childs, P. A. Conductance of Graphene Nanoribbon Junctions and the Tight Binding Model. *Nanoscale Res. Lett.* **6,** 1–5 (2011).

11. Ozaki, T., Nishio, K. & Kino, H. Efficient implementation of the nonequilibrium Green function method for electronic transport calculations. *Phys. Rev. B* **81,** 35116 (2010).

12. D'Agosta, R. Towards a dynamical approach to the calculation of the figure of merit of thermoelectric nanoscale devices. *Phys. Chem. Chem. Phys.* **15,** 1758–1765 (2013).

13. Abraham Fedorovich Ioffe. Energetic basis of thermoelectrical cells from semiconductors. *Acad. Sci. USSR, Moscow* (in Russia) (1950).

14. Dollfus, P., Nguyen, V. H. & Saint-Martin, J. Thermoelectric effects in graphene nanostructures. *J. Phys. Condens. Matter* **27,** 133204 (2015).

15. Gunlycke, D. & White, C. Tight-binding energy dispersions of armchair-edge graphene nanostrips. *Phys. Rev. B* **77,** 115116 (2008).

16. Soler, J. M. *et al.* The SIESTA method for ab initio order-N materials simulation. *J. physics. Condens. matter* **14,** 2745 (2002).

17. Perdew, J. P., Burke, K. & Ernzerhof, M. Generalized Gradient Approximation Made Simple. *Phys. Rev. Lett.* **77,** 3865–3868 (1996).

18. Monkhorst, H. J. & Pack, J. D. Special points for Brillouin-zone integrations. *Phys. Rev. B* **13,** 5188–5192 (1976).
